# Discovery of drug transporter inhibitors tied to long noncoding RNA in resistant cancer cells; a computational model -in silico- study

**DOI:** 10.3389/fimmu.2025.1511029

**Published:** 2025-04-25

**Authors:** Mohanad Diab, Amel Hamdi, Feras Al-Obeidat, Wael Hafez, Ivan Cherrez-Ojeda, Muneir Gador, Gowhar Rashid, Sana F. Elkhazin, Mahmad Anwar Ibrahim, Tarek Farag Ismail, Samar Sami Alkafaas

**Affiliations:** ^1^ Mediclinic Airport Road Hospital, Abu Dhabi, United Arab Emirates; ^2^ Molecular biology and Hematology, Abu Dhabi University, Abu Dhabi, United Arab Emirates; ^3^ College of Technological Innovation at Zayed University, Abu Dhabi, United Arab Emirates; ^4^ NMC Royal Hospital, Abu Dhabi, United Arab Emirates; ^5^ Department of Internal Medicine, Medical Research and Clinical Studies Institute, The National Research Center, Cairo, Egypt; ^6^ School of Health, Universidad Espíritu Santo-Ecuador, Samborondón, Guayas, Ecuador; ^7^ Respiralab Research Group, Guayaquil, Guayas, Ecuador; ^8^ Department of Clinical Biochemistry, Sher-i-Kashmir Institute of Medical Sciences (SKIMS), Srinagar, India; ^9^ Sheikh Khalifa Hospital, Al Fujairah, United Arab Emirates; ^10^ Molecular Cell Biology Unit, Division of Biochemistry, Department of Chemistry, Faculty of Science, Tanta University, Tanta, Egypt

**Keywords:** chemoresistance, p-glycoprotein-1, lncRNA, efflux transporters, in silico analysis

## Abstract

Chemotherapeutic resistance is a major obstacle to chemotherapeutic failure. Cancer cell resistance involves several mechanisms, including epithelial-to-mesenchymal transition (EMT), signaling pathway bypass, drug efflux activation, and impairment of drug entry. P-glycoproteins (P-gp) are an efflux transporter that pumps chemotherapeutic drugs out of cancer cells, resulting in chemotherapeutic resistance. Several types of long noncoding RNA (lncRNAs) have been identified in resistant cancer cells, including *ODRUL*, *MALAT1*, and *ANRIL.* The high expression level of *ODRUL* is related to the induction of *ATP-binding cassette (ABC)* gene expression, resulting in the emergence of doxorubicin resistance in osteosarcoma. lncRNAs are observed to be regulators of drug transporters in cancer cells such as *MALAT1* and *ANRIL.* Targeting P-gp expression using natural products is a new strategy to overcome cancer cell resistance and improve the sensitivity of resistant cells toward chemotherapies. This review validates the inhibitory effects of natural products on P-gp expression and activity using in silico molecular docking. *In silico* analysis showed that Delphinidin and Asparagoside-f are the most significant natural product inhibitors of p-glycoprotein-1. These inhibitors can reverse multi-drug resistance and induce the sensitivity of resistant cancer cells toward chemotherapy based on *in silico* molecular docking. It is important to validate that pre-elementary docking can be confirmed using *in vitro* and *in vivo* experimental data.

## Graphical Abstract

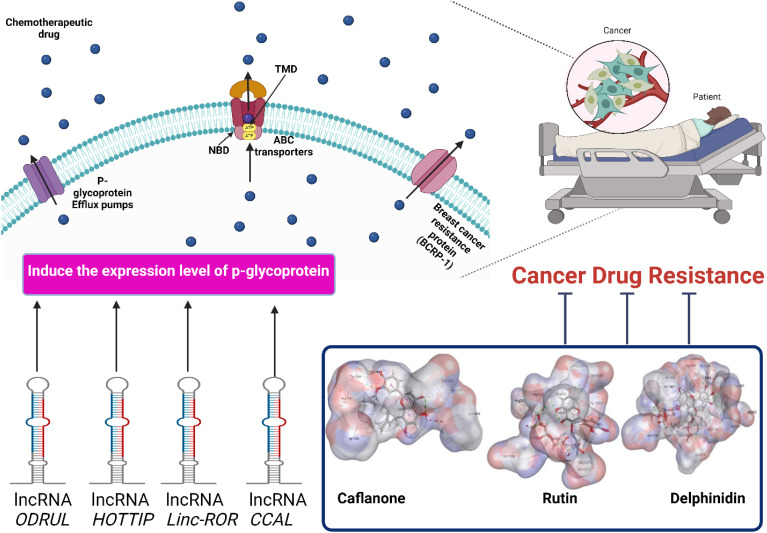

## 1 Introduction

Although there are many significant cancer treatments, many issues reduce the sensitivity and responsiveness toward chemotherapeutic drugs. There are many mechanisms for controlling cancer cell resistance and emergence of multidrug resistance, including overexpression of ABC transporters and p-glycoproteins (1). High expression levels of ABC transporters require ATP to efflux chemotherapeutic drugs from cells (2). P-glycoprotein (P-gp) is an efflux protein that is the main cause of Multidrug resistance (MDR) (3, 4). Juliano et coworkers observed P-gp expression on the surface of ovarian cells (5). ABCB1 encodes P‐gp, which consists of four domains: two nucleotide-binding domains and two transmembrane domains (6). P-gp is expressed at high levels on the surfaces of several cancers, including lung cancer, breast cancer, colon cancer, osteosarcoma, and hepatocellular carcinoma (7–9;MA et al., 2019;^10^, ^11^). P-gp transporters efflux chemotherapeutic agents from the cell, resulting in cancer cell resistance (12, 13). The chemotherapeutic substrates for P-gp transporters include paclitaxel, 5−fluorouracil, doxorubicin, and 5−fluorouracil (14, 15). Several studies have shown that lncRNAs are strongly associated with the emergence of multidrug resistance in several cancer cell types (16). Although lncRNA transcripts are > 200 nucleotides long, no protein-coding potential has been identified (17). Disturbances in lncRNA levels lead to chemoresistance in cancer cells (18, 19). lncRNAs regulate drug transporters in cancer cells, such as *MALAT1* and *ANRIL* which control the expression of Multidrug resistance protein 1 (MRP1) and Multidrug resistance gene 1 (MDR1) (20). Both *in vivo* and *in vitro* studies have shown that *MALAT1* is associated with the development of cisplatin-resistant A549 lung cancer cells. High expression levels of *ANRIL* induce cisplatin-resistant and 5-fluorouracil-resistant gastric cancer cells. In this context, inhibition of P-gp transporter expression decreases multidrug resistance and increases the responsiveness of resistant cancer cells toward chemotherapeutic drugs. Natural products can modulate cancer cell resistance by inhibiting P-gp transporter expression. P-glycoprotein is one of the chemoresistance mechanisms in cancer cells that causes long-term chemotherapeutic failure. lncRNAs, such as MALAT1, ANRIL, and ODRUL, are considered inducers of p-glycoprotein expression. Targeting P-gp expression is a significant strategy to overcome chemotherapeutic resistance and increase cancer cell sensitivity towards drugs. Because natural products are extracted from natural sources, they are considered favorable P-glycoprotein inhibitors without side effects. In this review, we describe several types of natural products that can increase the sensitivity of resistant cancer cells, which was confirmed by *in silico* molecular docking. In addition, this review describes the mechanisms of cancer drug resistance, different types of lncRNAs, and their relationship to chemotherapeutic resistance. This review presents an important step in the strategy to increase the responsiveness of resistant cancer cells; however, future *in vitro* and *in vivo* experiments are needed to confirm our preliminary docking results.

## 2 Mechanisms of cancer drug resistance

**Figure 1:**
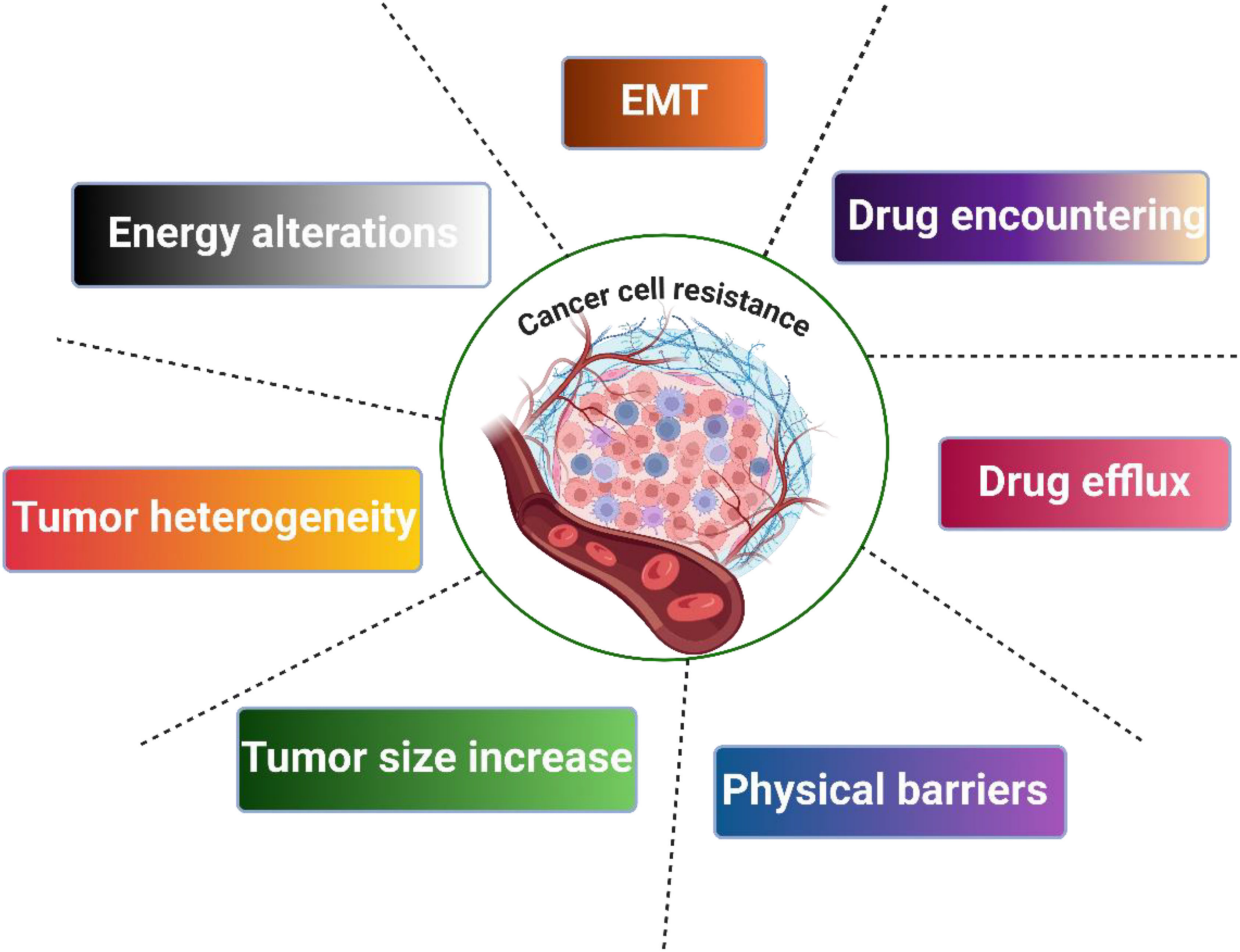
Mechanisms of cancer cell resistance.

Drug resistance can be classified into two classes: primary and secondary. Primary resistance appears before the exposure of cancer cells to chemotherapy. Secondary resistance arises from adaptation of cancer cells to chemotherapy. Drug resistance commonly results from genomic alteration. There are different resistance mechanisms, including p-efflux transporters, inhibition of drug entry, and EMT, as shown in **Figure 1** (21–24).

### 2.1 Tumor heterogeneity

Tumor heterogeneity is a significant factor underlying resistance to cancer drugs and is considered a fundamental feature of tumor progression and adaptation to different conditions. The evolutionary power of tumors is mainly linked to heterogeneity; colonies with more robust heterogeneity are favored during cancer progression and become dominant (25). Tumor heterogeneity can be attributed to both intrinsic and extrinsic factors. Generally, intrinsic factors are cellular in origin and can accumulate due to alterations in the levels of DNA, RNA, protein, epigenetics, and signal transduction. Genetic alterations include mutations, gene amplification, chromosomal aberrations, and miRNA gene changes. Alterations in transcriptomics, proteomics, and epigenetics could also originate from DNA alterations, which could modulate the cell cycle and its overall regulation (26). Additionally, modification of cancer stem cells (CSCs) supports heterogeneity, tumor plasticity, and resistance (27). Downregulation of pro-apoptotic molecules and upregulation of anti-apoptotic players participate in heterogeneity-associated resistance (28). Moreover, differences in signaling contribute to heterogeneity in cancer drug resistance heterogeneity (29). Tumors could possess a high level of stochasticity due to *de novo* differences in enzymatic signal transduction cascades that promote biological noise and subsequently develop feedback inhibition motifs to decrease biological noise (30).

Conversely, extrinsic or microenvironmental factors can promote heterogeneity and resistance through spatial differences in cells, blood supply, pH, hypoxia, and paracrine signaling (31, 32). Additionally, the vascular network and contact with cancer cells are arbitrary, resulting in fluctuations in the nutrient and metabolic status of different cancer cells (33).

### 2.2 Tumor burden and physical barriers

There is a significant association between tumor size and tumor resistance, and tumor size could be a determinant of tumor capacity to develop drug resistance mutations. Tumor growth and response to therapy have an inverse relationship with the growth rate (34). However, cancers can reveal spatial gradients that limit the blood supply and oxygen enrichment, creating an isolated hypoxic pro-tumorigenic environment with low contact with chemotherapy (34).

### 2.3 Tumor microenvironment

Tumor microenvironment (TME) interactions with tumor cells can aid in resistance. Tumors are not fully homogeneous but include different classes of cells and extracellular matrix (ECM), such as immune and inflammatory cells, fibroblast blood vessels, multiple nutrients, and signaling molecules (35). TME could be attributed to the pharmacological outcomes that force the cells to adapt to chemotherapy. One TME factor is pH; tumor cells typically exhibit a reversed pH gradient, with intracellular pH higher than extracellular pH, which promotes resistance to chemotherapy (36). Furthermore, alkaline pH modulates ion trapping, reduces drug efficacy, and helps cancer cells avoid apoptosis, promoting cell proliferation, tumor aggressiveness, invasion, and resistance to the immune response (37). Cycles of hypoxia, reoxygenation, and lack of O_2_ produce reactive oxygen species (ROS) that are associated with heterogeneity and resistance (38). In addition, EMT and CSCs have carcinogenic effects by helping cancer cells avoid apoptosis (39). Additionally, resistance could be attributed to TME attenuation of the immune clearance of cancer cells, and the TME could enhance resistance by inducing paracrine growth factors to mediate cancer cell growth (40). Chemotherapy pressure forces cells to possess a robust phenotype against stress (41). In addition, external pressure can modulate the expression of anti-apoptotic markers and epithelial-to-mesenchymal transition (EMT).

### 2.4 Cancer stem cells

The presence of stem cells in cancer tissues is linked to the resistance of many cancers, such as a long lifetime, high expression of drug exporters, elevated DNA repair mechanisms, and attenuated apoptosis (42). Stem-cell-dependent resistance is generally dependent on EMT machinery (43).

### 2.5 EMT

The EMT process is distinguished by the loss of both cell-cell contacts and apico–basal polarity associated with epithelial cells, ultimately acquiring mesenchymal features (44). The EMT program is mainly initiated by TME paracrine signaling by fibroblasts, macrophages, or immunocytes (44). EMT permits cancer cells to possess the ability to resist anticancer drugs and avoid apoptosis. EMT is characterized by elevation of transforming growth factor-beta (TGF-β), which significantly aids in resistance (45). EMT-linked transcription factors, including Twist1, Snail, Slug, ZEB, and FOXC2, are associated with drug resistance in cancer (46). These transcription factors support resistance by promoting drug efflux, such as ABC transporters, in addition to avoiding apoptosis via an immune response that shares similarities with the resistance profile of stem cancer cells (47–49). The EMT also has the capacity to self-renew and escape apoptosis. EMT is the initial step in escape from neighboring tissues and subsequent metastasis (50).

### 2.6 Drug manipulation

Drug efflux machinery, such as ABC transporter and efflux pump P-glycoprotein (P-gp), are enhanced through EMT, stem cells, miRNAs, and as a response to pharmaceutical pressure (51). Generally, drug uptake into cells occurs through diffusion through the plasma membrane (PM), transporter activity, and endocytosis. During cancer development, alterations are accompanied by changes in the lipid composition of the PM, such as phosphatidylserine (PS). In cancer cells, PS is exposed to an extracellular environment opposite to normal PM, which gives the cell more negative charge-altering drug entry (52, 53). Additionally, the attenuated pH of the extracellular media of cancer cells affects the ionization status of drugs and their entry (54). Drug entry is also dependent on several transporters called carriers (SLC), such as OATP1B3 and OCT6, which were found to be attenuated during treatment with doxorubicin and cisplatin (55). Moreover, elevated rigidity of endosomes affects the endocytosis process, thereby affecting drug entry (56, 57). In addition, alterations in drug targets, such as protein mutations or expression aberrations, suppress the robustness of targeted therapy. For instance, a missense variant in the epidermal growth factor receptor (EGFR) subsequently impairs the binding of gefitinib/erlotinib to the kinase (58).

### 2.7 Epigenetic alterations

Epigenetic modifications include methylation, histone modifications, and non-coding RNAs disturbances (59). Oncogene promoters can be demethylated and subsequently gene expression is elevated, as observed in several genes, including ID4, ERp29/MGMT, ETS-1,

and miR-663, which are involved in breast cancer resistance against several chemotherapeutics (60, 61). Similarly, the MDR1 and PD-L1/DNMT1 axes are hypomethylated in HCC cells treated with Doxorubicin and sorafenib, respectively (62). However, some gene promoters are hypermethylated, causing attenuation of gene expression and subsequent resistance, such as TGBI and ER-α, in breast cancer when Trastuzumab and Antiestrogen are administered, respectively (63, 64). Moreover, target genes and export pump functions can be enhanced by histone demethylation and acetylation (65). Furthermore, miRNA alterations that affect gene expression are involved in drug cancer resistance. For instance, miR-15b promotes resistance to cisplatin by targeting PEBP4- and RKIP-mediated EMT, similar to miR-27a (66). In addition, lncRNAs are overexpressed and promote proteins related to cancer drug resistance (67).

### 2.8 DNA damage repair

The DNA damage repair (DDR) machinery is controlled by several genes that are enhanced during cancer therapy, leading to resistance. Thus, impairment of the DDR can increase pharmaceutical sensitivity (68). O-6-Methylguanine-DNA Methyltransferase (MGMT) is responsible for the clearance of alkyl adducts from the O6 position of guanine; inactivation of this machinery was found to be a therapeutic target for sensitizing cells to O6-alkylating agents (69). DNA-dependent protein kinase (DNA-PK) is part of the double-strand break repair machinery (DSBs), and inhibition of this mechanism could promote radio/chemosensitivity of cancer cells (70).

### 2.9 Cell cycle

Irreversible cell arrest “senescence” could be provoked by several factors, including oncogenic genetic alterations, telomere erosion, and DNA damage linked to pharmaceutical therapy. However, the surviving cell populations may be more vigorous and highly proliferative (71). However, some cancer cells evade irreversible cell arrest and “senescence” by modifying apoptotic pathways to promote chemoresistance (72).

### 2.10 Energy alterations

Cancer cells develop characteristic metabolic phenotypes, especially glycolysis, known as the Warburg effect, which allows cancer cells to possess significantly higher intracellular ATP levels than normal cells of the same origin (73). Interestingly, cancer cell chemoresistance is correlated with ATP levels, and attenuation can sensitize cells (74). Increased cytosolic ATP levels are accompanied by elevated mitochondrial ATP levels in cancer- resistant cells. This phenomenon promotes drug efflux through ABC transporters, which in turn increases drug resistance (75). In contrast, the presence of extracellular ATP (eATP) in tumor cells is remarkably higher than that in normal cells, which is attributed to the elevated ATP produced from apoptosis and autophagy during therapy (76). Firstly, eATP can be transported to cells to promote resistance, as discussed earlier. Additionally, eATP signaling can promote EMT, cell growth, survival, and proliferation (77).

## 3 Drug efflux variations

49 of ATP-binding cassette transporters efflux chemical drugs from cancer cells, resulting in multidrug resistance (MDR). P-glycoprotein (P-gp), multi-drug-resistant associate protein (MRP), and adenosine triphosphate-binding cassette superfamily G member 2 (ABCG2) are the most common efflux transporters in ovarian and breast cancers (78). It has been observed that high expression levels of P-gp transporters in colorectal cancer and neuroblastoma lead to poor prognosis (79). P-gp transporters are encoded by the gene (MDR1) during the transformation of normal tissues into neoplastic tissues (80). Downstream receptors and proteins GTPase H-Ras, Mitogen-activated protein kinase 1/2 (MEK1/2), and Raf- 1 are involved in the mitogen-activated protein kinase (MAPK) pathway associated with high P-gp expression levels. On the other hand, Katayama, Imai, and their co-workers observed that inhibition of the extracellular signal-regulated kinase (ERK) pathway downregulates the expression level of P-gp (81, 82).

## 4 Effect of P-glycoprotein in tumor immunity

The high expression level of P-gp in immune cells induces their activation, modulation of their activity, and the release of cytokines. In the peripheral circulating system, the number of monocytes is very low, on the other hand, it increases in tissue tumor-infiltrating macrophages (83). The expression of P-gp in dendritic cells depends on its activation with a professional antigen (84, 85). Lloberas and his co-workers observed that using Valspodar to block P-gp prevented the maturation of dendritic cells and their activation markers CD80 and CD40 (85). Natural killer (NK (cells have a high P-gp expression level. There is a strong relationship between P-gp expression and the cytotoxic effects of NK. The high expression level of P-gp downregulates the cytotoxicity of NK cells by increasing the binding of Fas-mediated (Fas/FasL) P-gp+ NK cells to target cells. This triggers apoptosis of target cells by inducing the release of secretory granules with an inflammatory cytotoxic effect (86). In adaptive immunity, individual cell types determine the role of p-g expression. For example, in lymph nodes, the migration and transitional phenotype of B cells depends on the expression level of P-gp (87, 88). In CD4^+^T cells, Th1 and Th17 are effectors of T cells, and their inflammatory effect is associated with the expression level of P-gp. In contrast, the anti-inflammatory effect of T regulatory cells (Treg) limits the expression level of P-gp (89, 90). Kooij and his co-workers declared that memory (IL18Rα^+^CD161^+^CD62L^lo^) phenotype in CD8^+^T cells determines the expression level of P-gp (91). Bidirectional responses of P-gp expression were observed in CD8^+^ memory T cells in mucosal cells. In mucosal cells, P-gp normally effluxes xenobiotic toxins out of the cell; however, if a normal microbiome is distributed, this leads to enhanced effector responses and causes the emergence of autoimmune diseases such as Crohn’s disease. In acute myeloid leukemia, immune cells, including follicular lymphoma and B-cell lymphoma, boost P-gp expression levels, leading to chemotherapeutic resistance (92, 93). High MAP kinase/ERK signaling is associated with the induction of P-gp expression, which has a significant role in resistant myeloid leukemia (94). Ling et co-workers observed high P-gp expression levels in CD8+T cells derived from human colorectal cancer (95). In addition, chemoresistance in AML patients results from long-term chemotherapy and is correlated with the expression of CD4+CD161+P-gp+ T cells (96). On the other hand, Th17 and Th1 CD4+T-helper cells have been observed to trigger cytokine secretion, such as TNFα, and IL-17 which have anti-cancer activity (97). In this context, breast cancer cells have CD4+T-cells (CD4+CD73+T cells) that express P-gp and enhance the secretion of inflammatory and anticancer cytokines (98, 99). There is a conflicting role for P-gp in cancer cells, which is expressed in the pro-tumor effect of MΦ2-macrophage and the anti-cancer effect of NK-cell and Th17/CD4+T cells. Therefore, it is important to study the role of P-gp in immune cells.

## 5 lncRNAs related to p-glycoprotein

**Figure 2:**
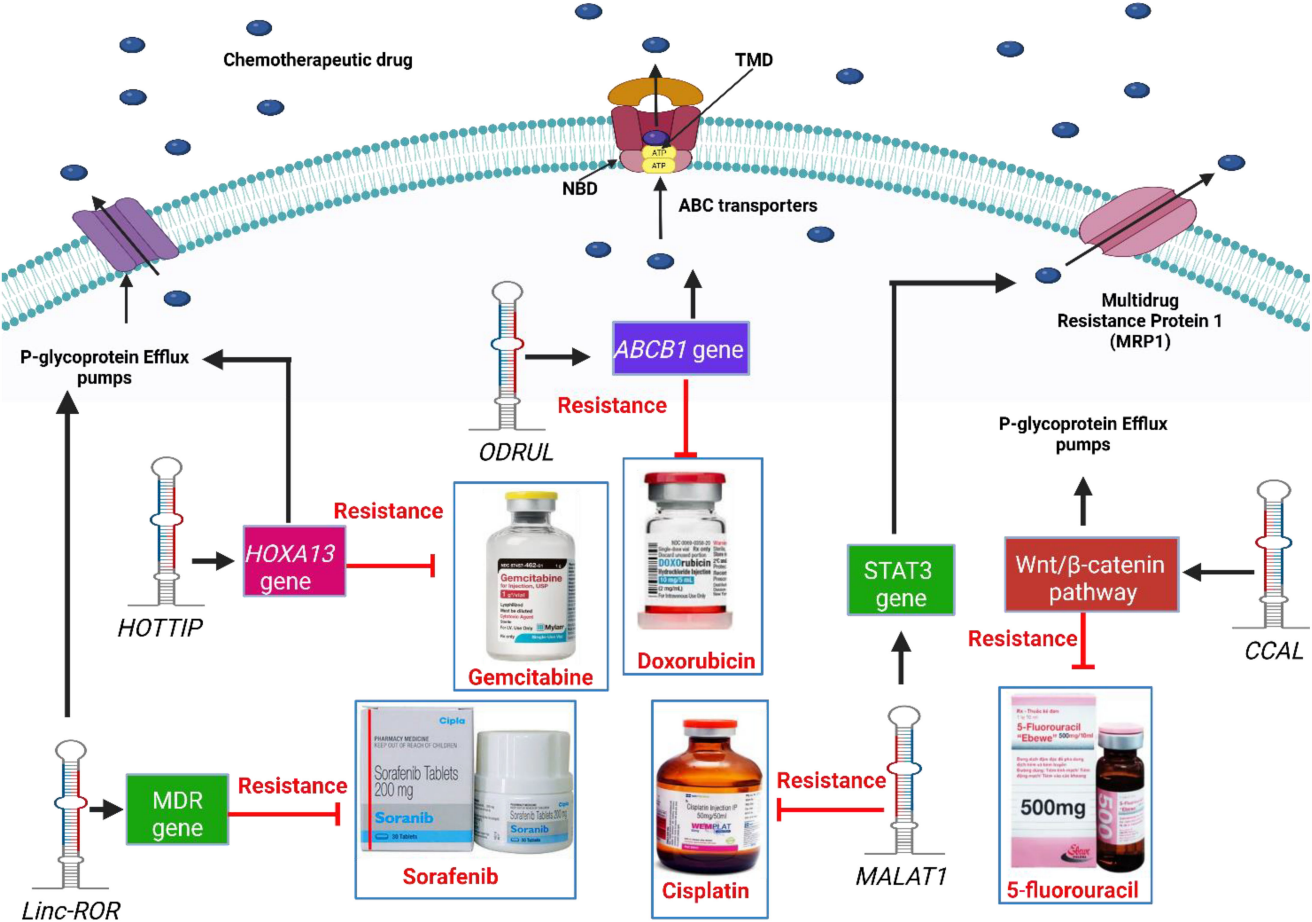
Mechanisms of lncRNAs related to drug efflux proteins which lead to chemoresistance.

Several studies have shown that lncRNAs play a significant role in increasing the expression levels of P-gp transporters and inducing multidrug, as shown in **Figure 2**. *ODRUL* was observed to be highly expressed in osteosarcoma cell lines. The high expression level of *ODRUL* is related to the induction of *ABCB1* gene and results in the emergence of doxorubicin resistance in osteosarcoma (100). Knockdown of the expression level of ODRUL leads to a decrease in the expression level of *ABCB1* gene and improves the responsiveness of osteosarcoma toward doxorubicin (100). In addition, high expression levels of lncRNA *HOTTIP* have been observed in resistant pancreatic ductal adenocarcinoma (PDAC) (101). *HOTTIP* was associated with gemcitabine-resistant PDAC cells. *In vitro* and *in vivo* studies showed that *HOTTIP* induces proliferation, invasion, and gemcitabine resistance in cancer cells by modulating *HOXA13* gene (101). *HOTTIP* knockdown improves the sensitivity of cancer cells toward gemcitabine (101). It was also observed that *H19* mRNA in resistant HepG2 cells induces the expression of p-glycoprotein transporters. High levels of *H19* mRNA induce doxorubicin resistance in HepG2 cells (102). Knockdown of *H19* mRNA induces the responsiveness and sensitivity of resistant cancer cells toward doxorubicin by increasing its accumulation and toxicity in both resistant and normal hepatoma cancer cells (102). Furthermore, knockdown of *H19* mRNA induces methylation of *MDR1* and then decreases P-glycoprotein expression (102). *Linc-ROR* lncRNA is upregulated in sorafenib-resistant HCC cells towards sorafenib. Knockdown of *linc-ROR* induces sorafenib toxicity and cancer cell death (103). High expression levels of CCAL lncRNAs induce multidrug resistance in colorectal cancer cells (104). *CCAL* lncRNA triggers a decrease in the signaling AP-2α protein-activated Wnt/β-catenin pathway, leading to upregulation of p-glycoprotein expression (104). On the other hand, low expression levels of snaR induce chemotherapeutic resistance in colon cancer toward 5-fluorouracil (105). High snaR expression induces apoptosis in colon cancer cells (105). High expression levels of *HOTAIR* induce platinum resistance in ovarian cancer cells. A high level of *HOTAIR* repairs the DNA damaged by platinum therapy, which activates NF-κB signaling, resulting in chemoresistance (106). Fang et co-workers observed that a high expression level of *MALAT1* is observed in cisplatin-resistant lung cancer. *MALAT1* induces cisplatin efflux from cells through efflux transporters (MDR1 and MRP1) after STAT3 activation (20). Furthermore, *ANRIL* was observed to induce the expression of efflux transporter proteins in resistant gastric cancer cells. Efflux transporter proteins pump cisplatin and 5-fluorouracil (5-FU) from cells, resulting in chemoresistance in gastric cancer cells (107). *ANRIL* knockdown increased the responsiveness of resistant cancer cells toward chemotherapeutic drugs by decreasing the levels of transporter proteins (107). *MRUL* increases the expression of *ABCB1*, which induces multidrug resistance. *ABCB1* gene is associated with efflux transporter proteins that pump doxorubicin out of cancer cells (108). *ABCB1* knockdown reduces drug efflux, leading to drug accumulation, toxicity, and apoptosis (108). The high expression levels of *linc-VLDLR* is implicated in the expression of the ABCG2. Chemotherapeutic drugs, including doxorubicin, sorafenib, and camptothecin, induce the expression of *linc-VLDLR* in both inside cells and in extracellular vesicles (EVs). Knockdown of *linc-VLDLR* reduces the level of ABCG2 drug efflux transporters and decreases cancer cell proliferation (109). lncRNA XIST controls the expression of Serum- and Glucocorticoid-Regulated Kinase 1 (SGK1), which sponges miR-124, resulting in doxorubicin resistance in colorectal cancer cells (110). Knockdown of *XIST* decreases the expression of p-glycoproteins and improved the responsiveness of resistant cancer cells toward DOX (110). Hu et co-workers observed that high expression levels of *KCNQ1OT1* are associated with oxaliplatin resistance in hepatoma cancer cells via the upregulation of efflux transporter genes, including MRP5, MDR1, and LRP1 (111). *KCNQ1OT1* knockdown decreases gene-related resistance and cancer cell growth, invasion, and migration. *A KCNQ1OT1* sponge with the 3′-UTR of miR-7-5p regulates the expression level of *ABCC1* mRNA in hepatoma cancer cells (111). *LINC00518* induces chemotherapeutic resistance in breast cancer cells by sponging miR- 199a (112). High levels of miR- 199a induce the expression of chemoresistant MRP1, resulting in paclitaxel, vincristine, and doxorubicin resistance in breast cancer. Knockdown of *LINC00518* expression level induces breast cancer cell sensitivity toward chemotherapy (112). Bladder cancer-associated transcript-1 (*BLACAT1*) is observed in resistant gastric cancer cells (113). A high expression level of *BLACAT1* is associated with oxaliplatin resistance in gastric cancer cells. *In vitro* and *in vivo* studies observed that knockdown of *BLACAT1* downregulates the expression of *ABCB1* protein and inhibits the proliferation of gastric cancer cells. miR-361 interacts with the 3′-UTR of *BLACAT1* and ABCB1mRNA resulting in chemoresistance (113). In conclusion, targeting p-glycoprotein and chemoresistance associated with lncRNAs is a new strategy for improving the responsiveness of resistant cancer cells toward chemotherapy.

## 6 Phytochemicals target both lncRNAs and p-glycoprotein to overcome chemoresistance

Several studies have reported that different types of natural products act as P-gp inhibitors (114). Natural products decrease chemoresistance by targeting p-glycoprotein expression (**Table 1**) (114). Natural products are characterized by having groups of methoxy, allyloxy, or acetylamino substituents, chiral configuration at C-3, and chromanol scaffolds, which can modulate the activity of p-glycoproteins (159). Baicalin and baicalein are natural products derived from the root of *Scutellaria baicalensis Georgi* that can downregulate P-gp expression in Caco-2 cells. Baicalin and baicalein showed significant anti-cancer activity, with IC_50_ = 479-332 μg/mL against Caco-2 cells. Miao et coworkers reported that *in vitro* studies of baicalein showed that baicalein has a greater inhibitory effect against P-gp than baicalin due to the presence of a glucosyl group (116). Chalcone is a natural phenolic compound that is extracted from apples, tomatoes, and licorice. *In vitro* studies have shown that chalcone inhibits p-glycoprotein activity, resulting in improved sensitivity of cancer cells toward chemotherapy (160). Cyanidin is a flavonoid extracted from leaves, vegetables, and fruits such as grapes, cherries, apples, beans, and cabbage. Cyanidin is cytotoxic to cancer cells by inducing apoptosis (161). Kitagawa observed that cyanidin decreased the expression levels of P-gp proteins and decreased chemoresistance (162). Quercetin is a flavonoid extracted from onion skin that has antioxidant activity. Quercetin inhibits chemotherapy transport by suppressing ATPase activity of ABCB1 (163). Rutin is a flavonoid extracted from the papaya plant. *In vitro* studies have shown that rutin inhibits P-gp activity and improves the responsiveness of cancer cells toward paclitaxel (123). Curcumin is a natural polyphenolic compound extracted from *Curcuma longa*. Curcumin inhibits the activity of P-glycoprotein regardless of the substrate formulation in LS180 Cells (164). Cinnamyl acetate is a natural phenylpropanoid compound extracted from the cinnamon bark. Cinnamyl acetate inhibits the expression of P-gp transporters and decreases chemoresistance in cancer cells (165). Hesperidin is a natural flavonoid extracted from citrus fruit. Kong et co-workers observed that hesperidin suppressed P-gp expression and induced the accumulation of chemotherapy in A549 cancer cells (166). Ursolic acid (UA) is a natural triterpene extracted from *Annurca* apples. Ursolic acid inhibits cancer cell proliferation by inducing apoptosis and upregulating caspase levels in resistant hepatoma cancer cells (167). Kaempferol is a flavonoid extracted from tea, curly kale, and blueberries. Kaempferol inhibits P-gp activity and decreases multi-drug resistance in KB-V1 cells (130). Luteolin is a natural product produced by *Helicteres hirsute.* Luteolin triggers cell death in cancer cells expressing efflux transporter proteins (ABCG2 and P-gp) by inducing ROS generation and DNA damage via inhibition of the NF-kB signaling pathway and downregulation of anti-apoptotic markers (134a). Sarsasapogenin is a steroid compound extracted from *Anemarrhena asphodeloides Bunge*. Sarsasapogenin suppresses the inflammatory activity that results from lipopolysaccharide (168). Fisetin is a flavone that is extracted from strawberries and apples. Kaempferol significantly inhibits P-gp expression more than fisetin, resulting in the efficient accumulation of daunorubicin (169). Glycyrrhizin is a natural product that is extracted from liquid plants. Glycyrrhizin inhibits P-gp activity and interacts with its substrate to inhibit P-gp transport (170). Noscapine is an alkaloid extracted from *Papaver somniferum.* Noscapine has been observed to have an inhibitory effect on P-gp, resulting in the suppression of multi-drug resistance in cancer cells (143). Allicin is a natural product that is extracted from garlic. Allicin can overcome p-glycoprotein and BCRP activity and induce the accumulation of sulfadiazine and florfenicol (171). Gingerol has also been extracted from *Zingiber officinale*. Gingerol inhibits P-gp activity and induces the accumulation of 3-H digoxin in Caco-2 cells (172). Gallocatechin gallate was extracted from green tea leaves. Gallocatechin gallate interacts with P-gp and decreases multidrug resistance in cancer cells (173). In conclusion, natural products can act as significant inhibitors to overcome multi-drug resistance and improve the sensitivity of cancer cells toward chemotherapy. Mondal et et al. observed that mahanimbine induced P-gp ATPase activity and decreased cancer cell resistance (174). Diindolylmethane is a dietary bioactive compound that modulates the efflux of ABC transporters and improves the efficacy of Centchroman in breast cancer cells (126). In addition, *in vitro* and *in vivo* studies have shown that betulinic acid downregulates the expression level of MALAT1 which is associated with hepatoma-resistant cells. In addition, bharangin is a natural product with a quinone- methide structure derived from *Pygmacopremna herbacea* (175). Studies have shown that bharangin downregulates the expression of H19 lncRNA in resistant breast cancer cells. Curcumin also reduces H19 lncRNA expression, which is associated with resistance in MCF-7 breast cancer cells (175). Curcumin affects EMT biomarkers, including N-cadherin and E-cadherin levels. It reduced the levels of N-cadherin and increased the levels of E-cadherin. *In vitro* studies have shown that curcumin decreases renal cancer cell migration and invasion by downregulating the expression of HOTAIR (124). Resveratrol is extracted from pistachios, plums, grapes, and berries (176). The expression level of MALAT1 is increased in resistant colon cancer cells. Resveratrol decreases colon cancer resistance by downregulating MALAT1 and mediating the Wnt/β-catenin signaling pathway. Silibinin decreases the expression of HOTAIR by modulating the PI3K pathway (177). Therefore, the inhibitory effect of the natural products was validated by *in silico* molecular docking analysis; however, future *in vitro* and *in vivo* experiments are needed to confirm our preliminary docking results.

**Table 1:** Phytochemicals list that target P-glycoprotein and or LncRNAs to overcome chemoresistance in different cancer cells.

Phytochemicals	Plant Source	Bioavailability	Concentration IC_50_	P-glycoprotein/LncRNAs	*In vitro*/*in vivo* experiments	Reference
Baicalein	Root of *Scutellaria baicalensis* Georgi (Labiatae)	The absolute bioavailability of baicalein in different doses ranged from 13.1% to 23.0%.	332 μg/mL	Baicalein inhibits the expression and activity of P-glycoprotein resulting in the accumulation of intracellular rhodamine 123. Baicalein down-regulates BDLNR in poor cervical cancer *in vivo* which is bound to Y-box binding protein 1 (YB-1), recruited YBX1 to PIK3CA promoter, activated PIK3CA expression and PI3K/Akt pathway.	Caco-2 cells, cervical cancer, and rat gut sacs	(115–117)
Baicalin	Root of *Scutellaria baicalensis* Georgi (Labiatae)	low bioavailability of about 2.2 ± 0.2%	479 μg/mL	Baicalin doesn’t affect P-glycoprotein activity. This is because of the structure-activity relationship of the inhibitors of P-gp. Baicalin has glucosyl that influences the activity of P-gp and downregulates its inhibitory effect.	Caco-2 cells and rat gut sacs	(116, 118)
Quercetin	Amaryllidaceae, Brassicaceae, Capparaceae, Ericaceae, Liliaceae, and Rosaceae.	The bioavailability of quercetin is relatively low (<10%)	0.044 mM	Quercetin targets MALAT1 and decreases invasion in prostate cancer by upregulating N-cadherin and phosphorylated Akt; downregulating E-cadherin.	Prostate cancer	(119–122)
Rutin	Buckwheat, apricots, cherries, grapes, grapefruit, plums, and oranges	Poor bioavailability	8 μM	Rutin is observed to inhibit P-gp transport function and significantly reduce resistance in cytotoxicity assay to paclitaxel in P-gp overexpressing MDR cell lines.	KB 3-1 and KB CH^R^ 8-5 cell lines	(123b)
Curcumin	*Curcuma longa*	Very poor bioavailability	0,5,15, and 20 M	Curcumin downregulates both H19 and HOTAIR in renal carcinoma and breast cancer cells. Curcumin affects EMT biomarkers including N-cadherin and E-cadherin levels. It reduces levels of N-cadherin and increases levels of E-cadherin. *In vitro* studies observed that curcumin decreases renal cancer cell migration and invasion by downregulating the expression level of HOTAIR (124)	MCF-7/TAMR * cell line. 769-P-HOTAIR and 786-0 cell lines	(125)
Diindolylmethane	cruciferous vegetables	DIM is poorly absorbed from the gastrointestinal tract	56.23 µM	Diindolylmethane induces intracellular accumulation of Hoechst and Calcein, the substrates of P-gp and MRP1, respectively, in breast cancer cells. In addition, Diindolylmethane induces P-gp ATPase activity and inhibits its efflux activity.	MDA-MB-231 cells Breast cancer cells	(126)
Fisetin	Strawberries, apples, grapes, onions, tomatoes, and cucumbers.	Low bioavailability (44.1%)	50 µM	Fisetin inhibits prostate cancer cell proliferation, migration, and invasion by modulating a P-glycoprotein overexpressing multidrug-resistant cancer cell line NCI/ADR-RES.	NCI/ADR-RES prostate cancer cell	(127–129)
Kaempferol	Leaves of *Ginkgo biloba*	Low poor at ~ 2%	43 μmol/L	Kaempferol increases the intracellular accumulation and reduces the efflux of Rh123 and ^3^[H]vinblastine in KB-V1 cells	Multidrug-resistant human cervical carcinoma KB-V1 cells	(130b;^131^, ^132^)
Luteolin	*Vitex negundo* leaves	The low bioavailability 4.10%	3–50 µM	Luteolin induces apoptosis in P-glycoprotein- and ABCG2-expressing MDR cancer cells without affecting the transport functions of these drug transporters.	BCRP-expressing MCF-7/Mito^R^ cells	(133, 134b;^135^)
Glycyrrhizin	licorice root	Approximately 1%	267.3 µM	Glycyrrhizin has anti-cancer and antioxidant activity. It reduces multidrug resistance (MDR) in cancer cells. Glycyrrhizin is a nitric oxide regulator in cancer cells and its subsequent anti-MDR effect.	Breast cancer cells HCT116	(136–138)
Gingerol	Rhizome of the ginger plant	Poor bioavailability	96.32 μM	It is observed that the exposure at 8 µM doxo concentrations in the presence of ginger improves drug accumulation and cytotoxicity on resistant MES-SA/Dx5 cells. Ginger induces the production of GSH content in resistant cells and decreases the multidrug resistance in resistant cells.	Human uterine sarcoma cell line MES-SA MES-SA/Dx5 cells	(139–141)
Noscapine	Papaveraceae, Berberidaceae, and Ranunculaceae	Bioavailability was 30%	34.7 μM	Noscapine minimizes endothelial cell migration in the brain by targeting endothelial cell activator interleukin 8 (IL-8). It modulates P-gp activity efflux on resistant cancer cells.	breast adenocarcinoma cell line MCF7	(142–144)
Anethole	Anise (*Pimpinella anisum*), fennel (*Foeniculum vulgare*), star anise (*Illicium verum*), basil (Ocimumbasilicum), tarragon (*Artemisia dracunculus*)	Limited bioavailability	50 µM	Anethole has multiple anti-cancer mechanisms, such as inducing apoptosis, causing cell cycle arrest, exhibiting anti-proliferative and anti-angiogenic effects, and modulating critical signaling pathways including NF-κB, PI3K/Akt/mTOR, and caspases.	Breast, prostate, lung, and colorectal cancers	(145, 146)
Procyanidin	Apples, maritime pine bark, cinnamon, cocoa beans, grape seed, grape skin, and red wines of *Vitis vinifera*	The bioavailability depends on their degree of polymerization. The absorption rate of proanthocyanidin dimers is 5–10%.	6.88 M	Procyanidin reverses MDR in A2780/T cells by inhibiting the function and expression of P-gp in A2780/T cells. Procyanidin reversed MDR by inhibiting the function and expression of p-gp via inhibition of NF-κB mediated by dephosphorylation of AKT and ERK1/2, respectively.	Ovarian cancer cell line A2780/T OAW42 and OVCAR3 cells	(147–149)
Allicin	Garlic cloves	The bioavailability of allicin is poor	10–25 μM	Allicin activates osteosarcoma immunoreactivity and induces apoptosis through the CBR3-AS1/miR-145-5p/GRP78 molecular axis. Allicin triggers silencing CBR3-AS1 led to reduced Saos-2 activity, enhanced apoptosis, and activated mitophagy and endoplasmic reticulum stress.	osteosarcoma	(150, 151)
Astaxanthin	Algae, yeast, salmon, trout, krill, shrimp and crayfish	Less than 10% for raw uncooked vegetables	<200 μM	RUSC1-AS1 is a novel oncogenic lncRNA in osteosarcoma through the miR-101-3p-Notch1-Ras-ERK pathway, which might be a potential therapeutic target for osteosarcoma. Astaxanthin down-regulates RUSC1-AS1 significantly attenuated the proliferative, epithelial-mesenchymal transition (EMT), growth, lung metastasis, migrative and invasive abilities of MG-63 and Saos-2 cells	Osteosarcoma MG-63 and Saos-2 cells	(152–154)
Dihydromyricetin	Leaves of grossedentata	Poor bioavailability	20.69 µg/mL	Dihydromyricetin effectively reversed multi-drug resistance occurring in SGC7901/5-FU cells cultured *in vitro* by downregulating MDR genes. It also decreased lncRNA MALAT1 expression which induces CSCC cell death via inducing excessive autophagy, which is mediated through the MALAT1-TFEB pathway.	SGC7901/5-FU cells Cutaneous squamous cell carcinoma (CSCC)	(155, 156)
Cinnamaldehyde	Bark, leaves, and twigs of various Cinnamomum species	The oral bioavailability of cinnamaldehyde is around 20%	9.48 and 9.12 μg/m	cinnamaldehyde increased the curative effect of oxaliplatin by promoting apoptosis both *in vitro* and *in vivo*. Cinnamaldehyde and oxaliplatin synergistically reversed hypoxia-induced EMT and stemness of CRC cells and suppressed hypoxia-activated Wnt/β-catenin pathway synergistically. It inhibits P-glycoprotein expression through inhibition of STAT3 and AKT signaling to overcome drug resistance	Lovo and HT-29 cells colorectal cancer (CRC)	(157, 158)

## 7 How specific lncRNAs regulate drug transporters like P-gp

The regulation of P-gp by lncRNAs typically occurs through various signaling pathways and mechanisms. Here’s a more specific look at how lncRNAs may regulate P-gp expression and function:

### 7.1 Transcriptional regulation

Some lncRNAs can regulate the transcription of the ABCB1 gene, which encodes P-gp, through various transcription factors. LncRNAs may act as scaffolds or guides for transcription factors or chromatin remodeling complexes to either promote or repress the expression of ABCB1 [1]. The lncRNA MALAT1 has been reported to influence the transcriptional regulation of drug resistance genes, including P-gp, in cancer cells. MALAT1 can interact with specific transcription factors and chromatin modifiers to enhance the expression of ABCB1, increasing the efflux of chemotherapeutic agents and promoting drug resistance [2].

### 7.2 Epigenetic regulation

lncRNAs can interact with chromatin-modifying complexes and enzymes to modify the chromatin structure and regulate the expression of P-gp through epigenetic mechanisms [3, 4]. This regulation often involves histone modifications or DNA methylation patterns at the ABCB1 gene locus. The lncRNA HOTAIR is known to regulate the expression of P-gp through epigenetic modifications. HOTAIR interacts with polycomb repressive complexes to silence genes that may inhibit the expression of P-gp, potentially increasing its activity in the context of drug resistance [4].

### 7.3 miRNA sponging

Many lncRNAs can act as miRNA sponges, sequestering specific microRNAs (miRNAs) that normally target the ABCB1 gene or its associated regulatory pathways. By binding to these miRNAs, lncRNAs prevent them from inhibiting the expression of P-gp [1]. The lncRNA H19 has been shown to sponge miR-675, which could normally suppress ABCB1 expression. By binding to miR-675, H19 indirectly promotes P-gp expression and drug resistance in certain cancers [5].

### 7.4 Interaction with signaling pathways

LncRNAs can modulate multiple signaling pathways, such as the PI3K/Akt, NF-kB, and MAPK pathways, which are known to regulate the expression of drug transporters like P-gp [6].

- **PI3K/Akt Pathway**: LncRNAs such as LncRNA-ATB have been shown to regulate the PI3K/Akt signaling pathway, which can enhance the expression of P-gp. This pathway is involved in cellular responses to stress and can influence drug transporter activity in cancer cells [7].
- **NF-kB Pathway**: Some lncRNAs, including LncRNA-MALAT1, may regulate the NF-kB signaling pathway, which is involved in inflammation and immune responses. NF-kB activation can also upregulate P-gp expression, particularly in the context of inflammation or drug resistance [8].

### 7.5 Post-transcriptional regulation

In addition to transcriptional regulation, lncRNAs can also affect P-gp at the post-transcriptional level. For instance, lncRNAs may modulate the stability of ABCB1 mRNA or influence its translation [1]. LncRNAs like TUG1 can regulate the stability of specific mRNAs through interactions with RNA-binding proteins, influencing the translation of P-gp and its levels in cells [1, 9].

### 7.6 Involvement in drug resistance mechanisms

lncRNAs are implicated in the development of drug resistance through their ability to modulate drug transporters like P-gp. For example, overexpression of certain lncRNAs can lead to increased P-gp activity, reducing the intracellular concentration of chemotherapeutic agents and thus contributing to resistance[10]. LncRNA-CCAT2 has been reported to be involved in chemoresistance by regulating the expression of P-gp. This lncRNA modulates the cellular response to chemotherapy drugs and enhances P-gp-mediated drug efflux [11].

## 8 Computational and preclinical studies of p-glycoprotein -1 in chemoresistance cancer cells

**Figure 3:**
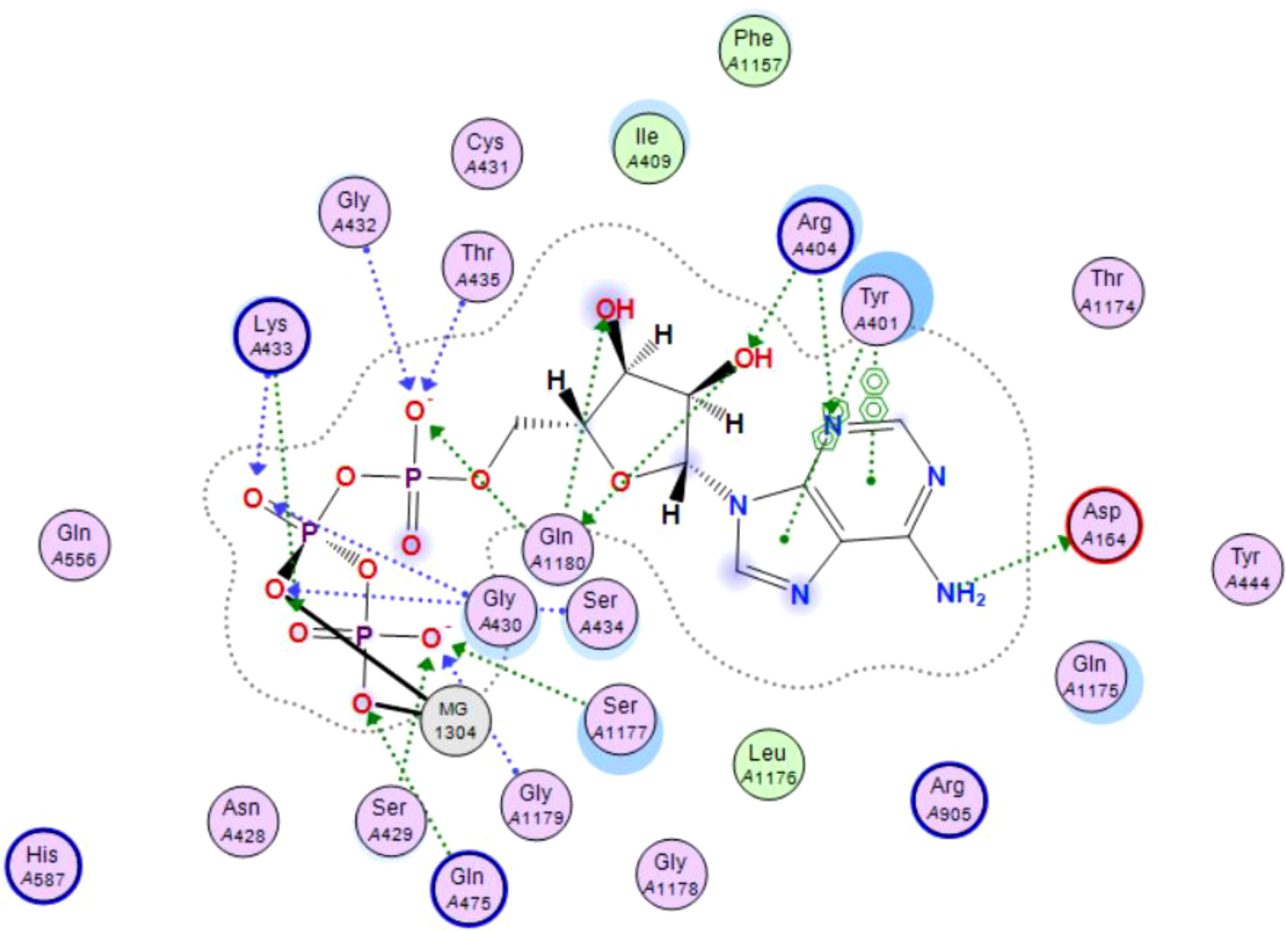
Co-crystallized ligand interacted inside active site.

In this review, we validate the potential effects of natural products against p-glycoprotein-1 to understand their association with cancer cell resistance. To perform molecular docking and illustrate inhibitor reactions, we used both the Molecular Operating Environment software (MOE, 2015.10) and BIOVIA Discovery Studio Visualizer (178). We followed the steps of a previously reported procedure to illustrate the reactions of inhibitors with significant amino acids or protein hotspots (178–180). The 3D structures of the targeted proteins were obtained from the Protein Data Bank (PDB). As shown in **Figure 3**, docking of the human P-glycoprotein in the ATP-bound, outward-facing conformation was performed using PDB 6C0V for p-glycoprotein -1 inhibitors. The exact binding site of bioactive compounds is the active site at which the co-crystallized ligand binds. All structure minimizations were conducted until an RMSD gradient of 0.05 kcal··mol^−1^Å^−1^ with *MMFF94x* force field, and partial charges were automatically calculated. Furthermore, all water molecules were removed from the compounds and p-glycoprotein-1 was prepared for docking using *the Protonate 3D* protocol in MOE with default parameters. To calculate both docking and scoring, we employed the *triangle Matcher placement* method and *London dG* scoring function. First, self-docking of the cocrystallized ligand near the protein- binding site was performed to ensure the docking protocol steps. Subsequently, ligand-receptor interactions at the target protein-binding site for the reported natural products in the active site were studied using a validated docking protocol (RMSD < 2) to predict their binding approach and binding affinity. The inhibitory activity of the tested substances was compared with that of the most potent p-glycoprotein inhibitor (mifepristone) through computational analysis. The plausible modes of binding between these substances and their target binding sites were determined. Delphinidin 3,5-di(6-acetylglucoside) (docking score: *S* = -10.0549 kcal/mol) was found to have the most significant inhibitory activity in the p-glycoprotein-1 inhibitor group (**Table 2**), with a higher potency than that of the control (mifepristone) (S = -5.1600 kcal/mol). Delphinidin 3,5-di (6-acetyl glucoside) interacts with the p-glycoprotein-1 active site via hydrogen bonds with the LEU ^531^ H-donor, GLN^535^ H-donor, ASP ^805^ H-donor, ASP ^805^ H-donor, SER ^1077^ H-acceptor, and TYR ^1044^ pi-pi. In addition, asparagoside-f (docking score: *S* = -9.0916 kcal/mol) was found to have the 2^nd^ highest inhibitory activity within the group, as shown in **Table 2**. Furthermore, Quercetin, caflanone, rutin, curcumin, kaempferol, and kazinol-f had more potent inhibitory effects than the control (mifepristone) (S = -5.1600 kcal/mol). Based on docking simulations, it can be concluded that these inhibitors can effectively inhibit p-glycoprotein-1 and are therefore considered potent drugs to treat chemoresistance or increase the responsiveness of cancer toward chemotherapy. Our molecular docking results represent the first step toward overcoming chemoresistance. In this context, *future in vitro* and *in vivo*, future experiments are required to confirm our results.

**Table 2:** Docking energy scores and amino acids involved in binding for Mifepristone, and the reported natural product inhibitors docked with the Molecular structure of human P-glycoprotein in the ATP-bound, outward-facing conformation (PDB: 6C0V).

Name	Amino acids involved in binding	CDocker energy (kcal/mol)	2D structure	3D structure
Co-crystallized ligand	GLN ^1180^ (A) H-donor ASP ^164^ (A) H-donor LYS ^433^ (A) H-acceptor SER ^429^ (A) H-acceptor SER ^1177^ (A) H-acceptor GLY ^1179^ (A) H-acceptor GLN ^475^ (A) H-acceptor GLY ^430^ (A) H-acceptor LYS ^433^ (A) H-acceptor SER ^434^ (A) H-acceptor GLY ^432^ (A) H-acceptor SER ^434^ (A) H-acceptor THR ^435^ (A) H-acceptor THR ^435^ (A) H-acceptor GLN ^1180^ (A) H-acceptor ARG ^404^ (A) H-acceptor ARG ^404^ (A) H-acceptor SER ^434^ (A) H-acceptor GLN ^475^ (A) H-acceptor GLN ^475^ (A) H-acceptor SER ^434^ (A) H-acceptor LYS ^433^ (A) ionic TYR ^401^ (A) pi-pi	-6.7843	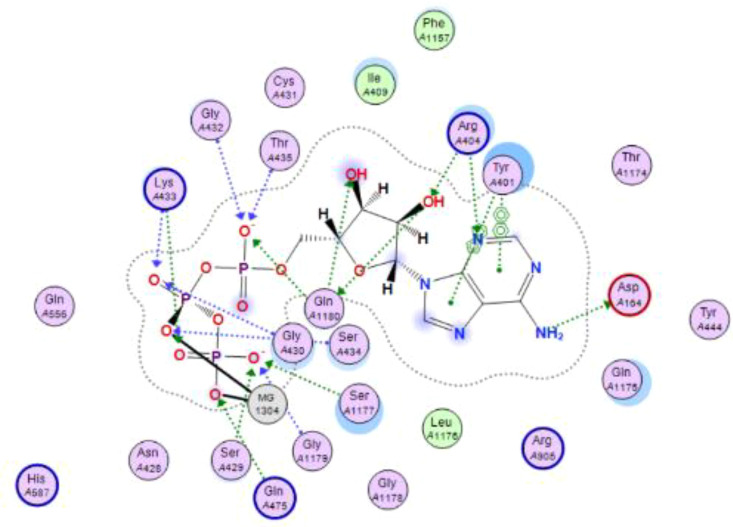	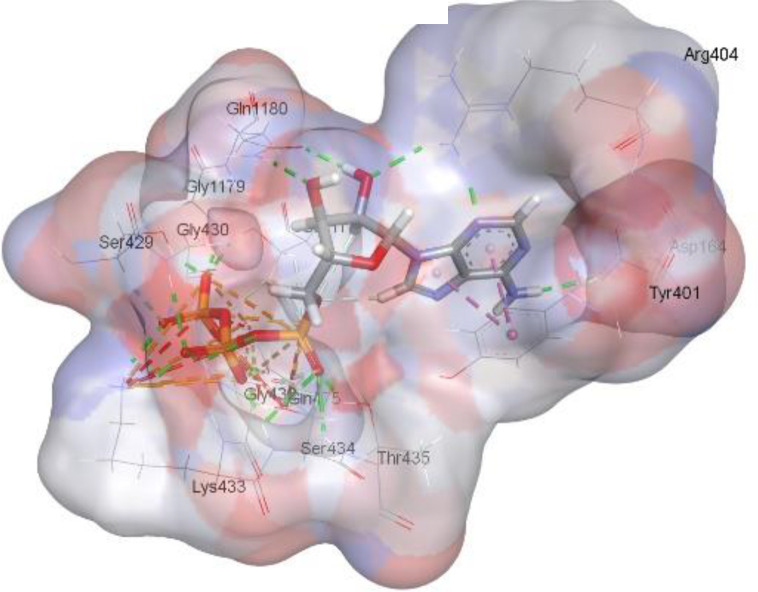
(Standard) Mifepristone The most significant p-glycoprotein-1 inhibitor	TYR ^401^ pi-pi	-5.1600	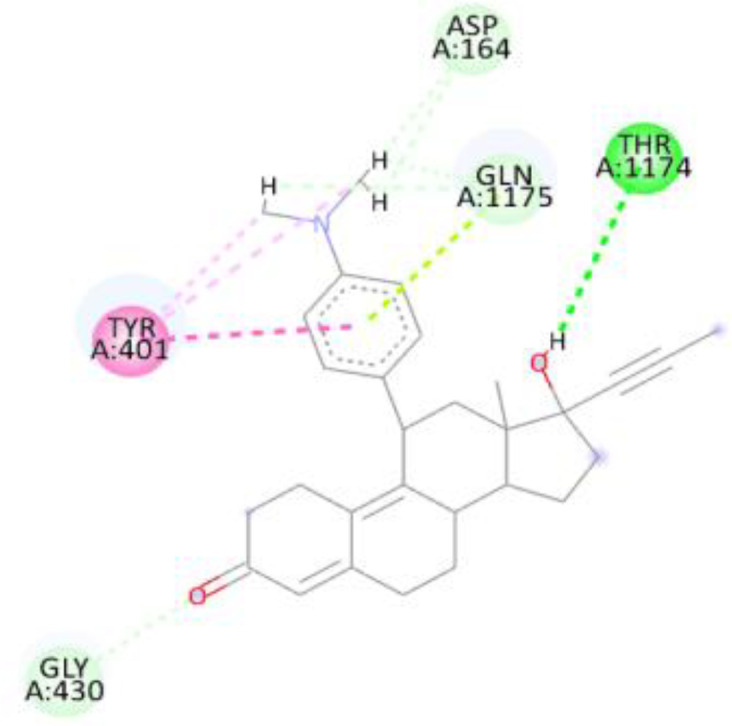	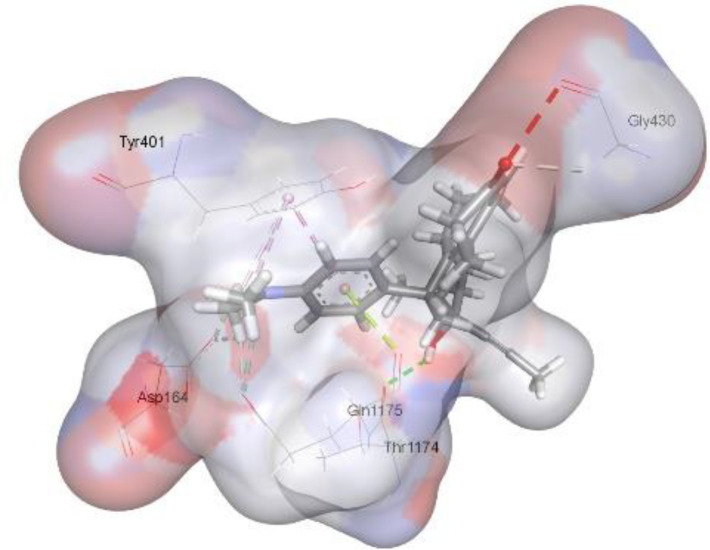
Baicalin	ALA ^529^ H-donor GLN ^1081^ H-acceptor ARG ^262^ H-acceptor ARG ^262^ ionic ARG ^262^ ionic	-4.5814	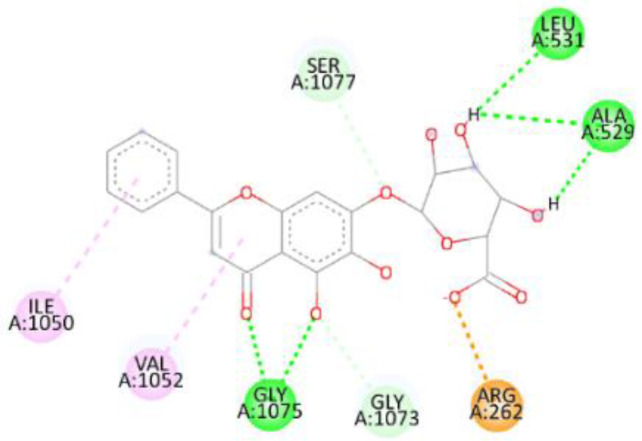	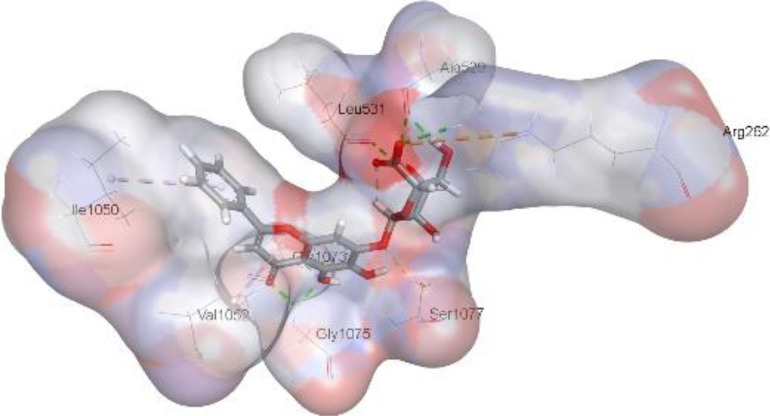
Baicalein	ASP ^164^ H-donor ASP ^164^ H-donor ARG ^905^ H-acceptor ARG ^905^ ionic ARG ^905^ ionic TYR ^401^ pi-pi	-6.2383	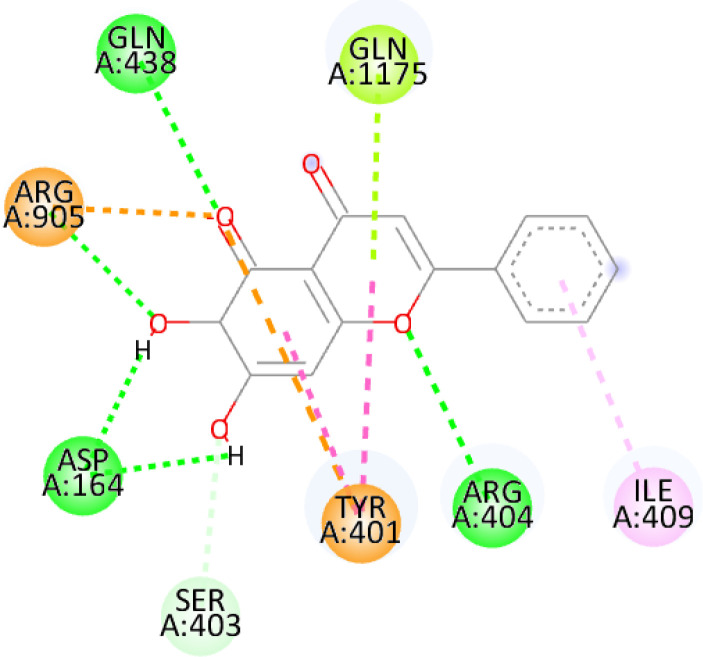	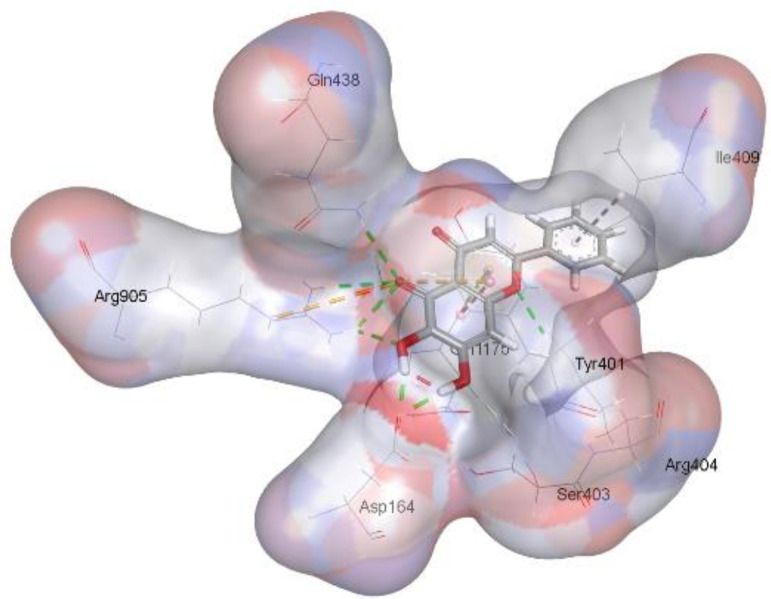
Caflanone	SER ^532^ pi-H TYR ^1044^ pi-pi	-7.0596	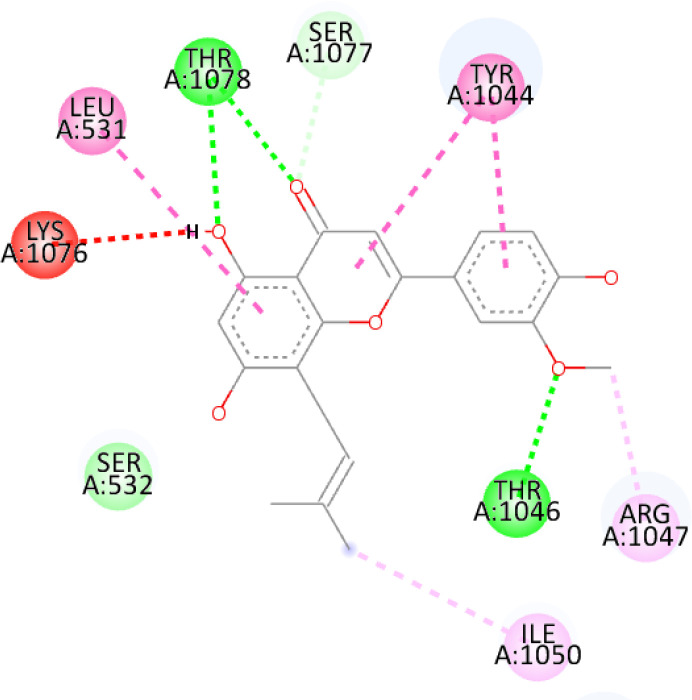	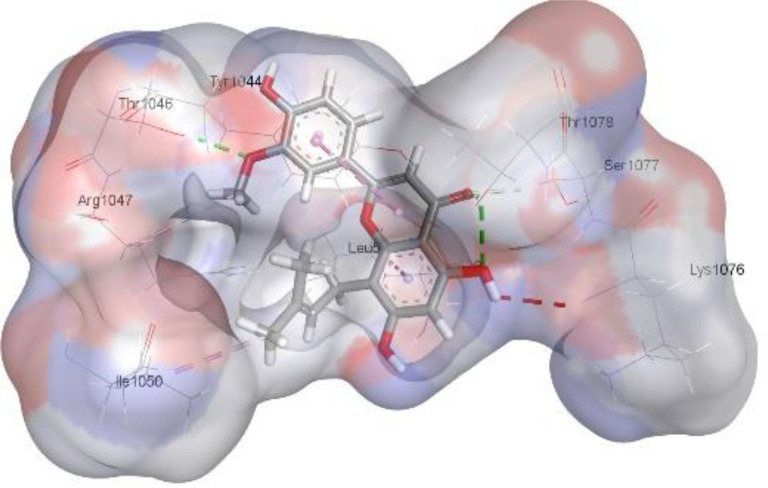
Cyanidin	ASP ^164^ H-donor ARG ^404^ H-acceptor ARG ^404^ H-acceptor ARG ^404^ ionic ARG ^404^ ionic TYR ^401^ pi-pi	-6.4466	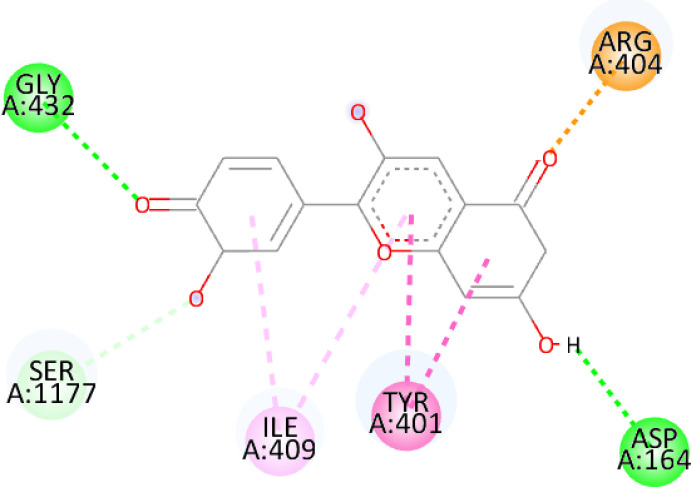	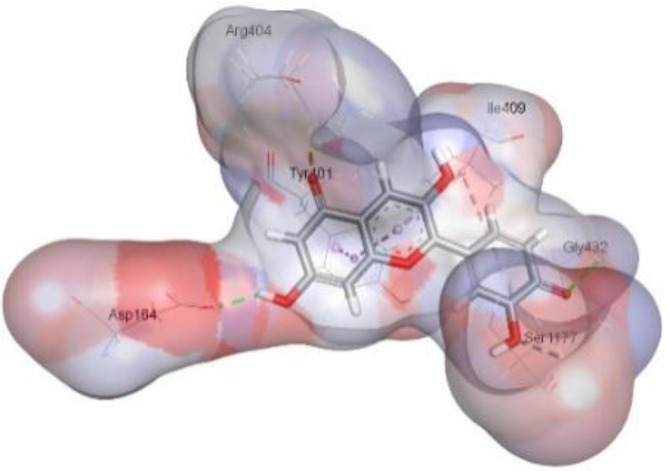
Quercetin	GLY ^533^ H-acceptor TYR ^1044^ pi-pi	-7.4963	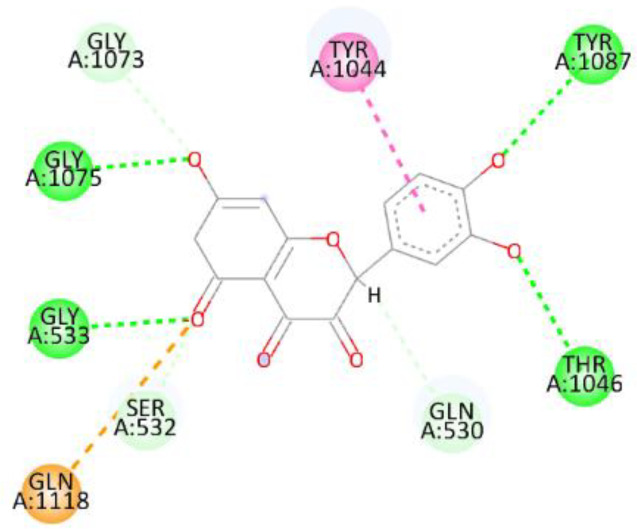	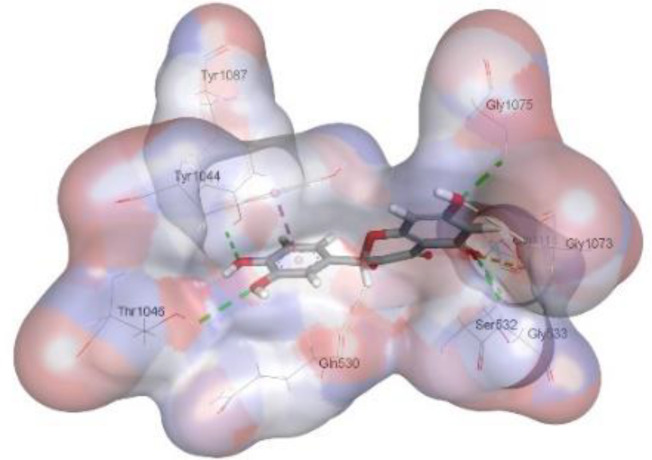
Rutin	ASP ^805^ H-donor LEU ^531^ H-donor SER ^532^ H-acceptor ARG ^262^ H-acceptor GLN ^530^ pi-H TYR ^1044^ pi-pi	-8.2013	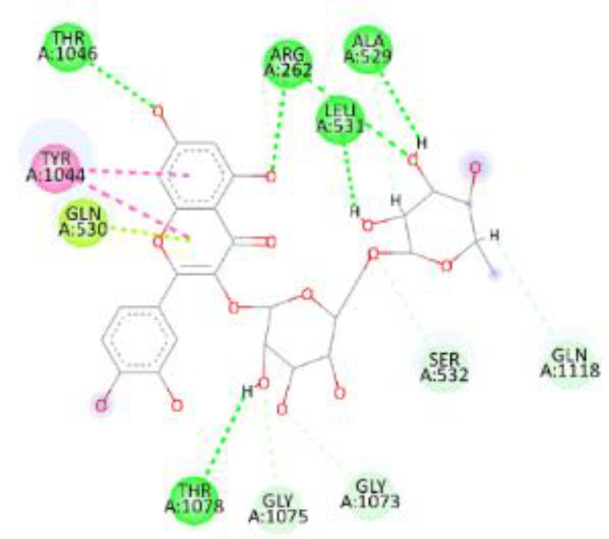	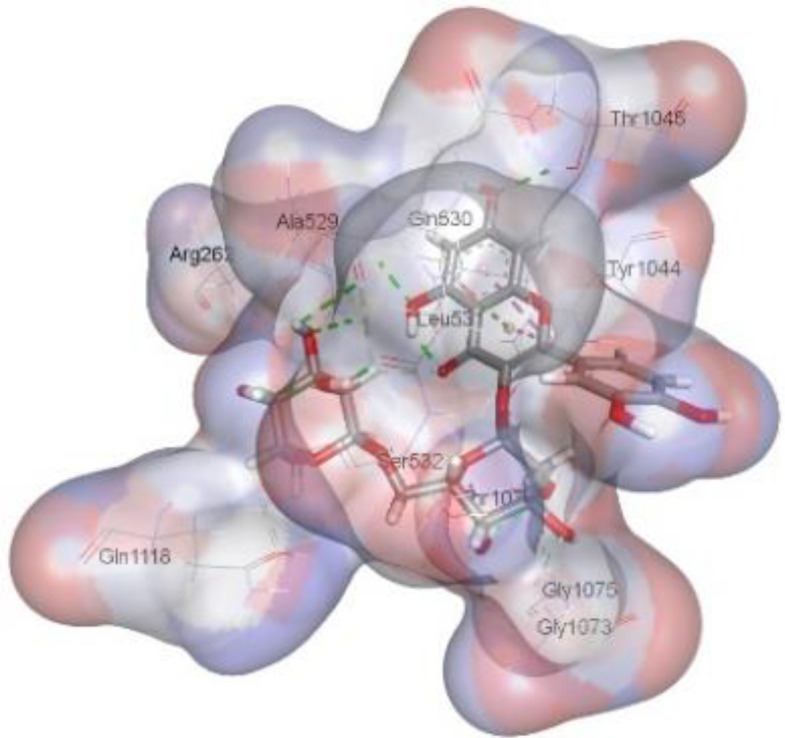
Digitoxigenin	TYR ^1044^ (A) pi-pi	-5.5567	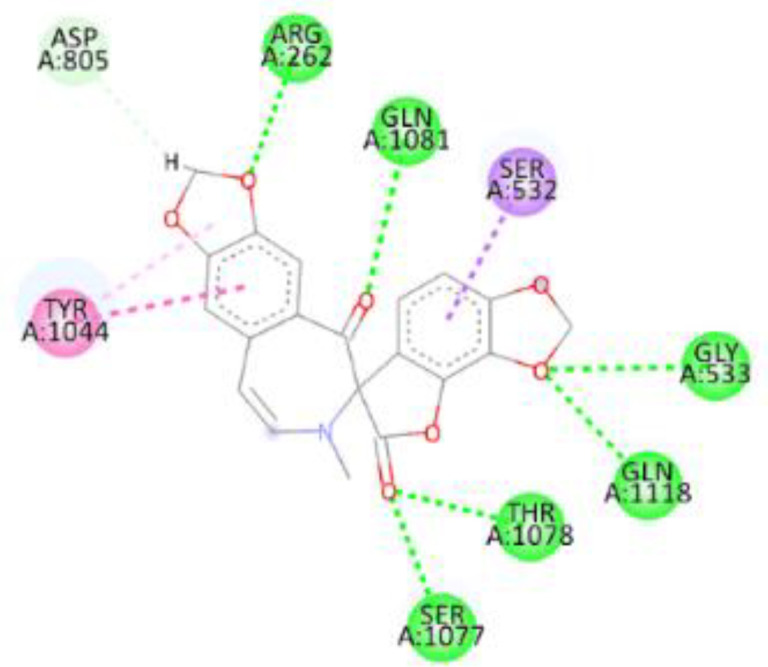	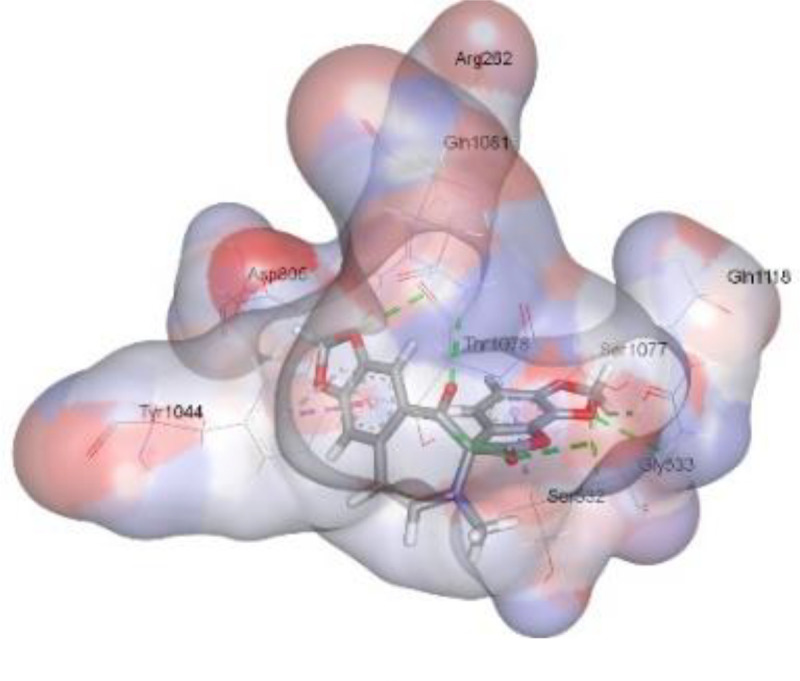
Curcumin	GLY ^430^ H-donor GLN ^438^ H-acceptor TYR ^401^ pi-pi	-7.1348	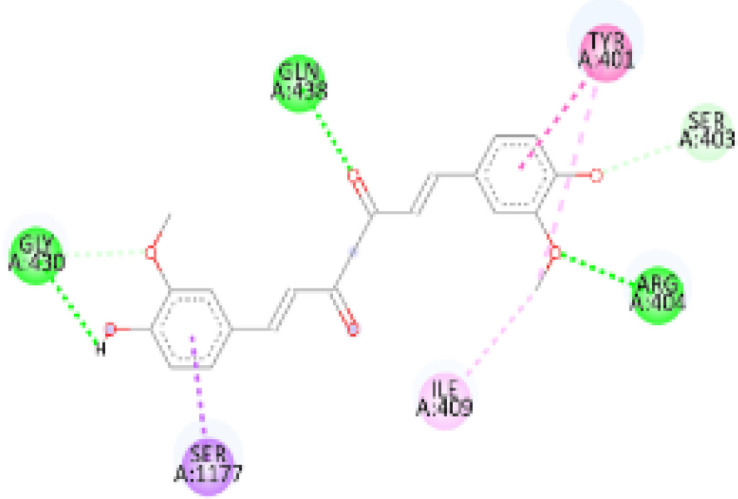	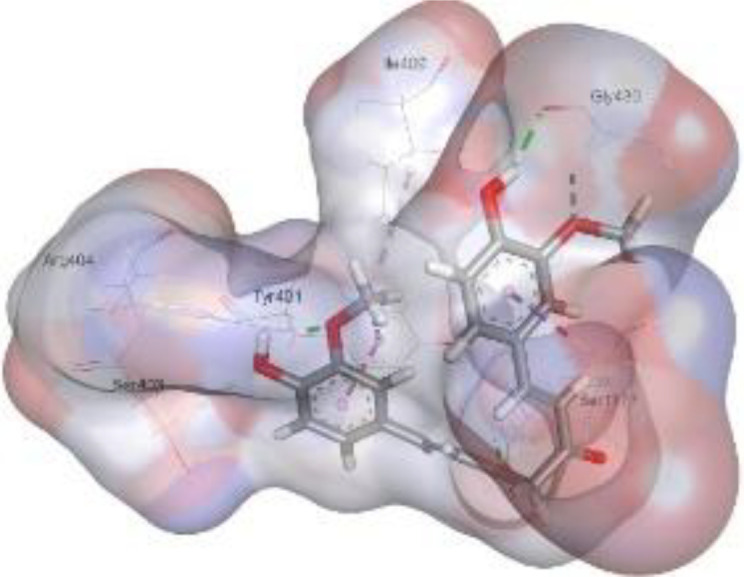

## 9 Conclusion

Improving cancer cell responsiveness is a significant step toward enhancing the efficiency of chemotherapeutic drugs. Resistance to chemotherapy is one of the main causes of chemotherapeutic failure. P-gp is a membrane transporter that causes efflux of drugs from cancer cells and results in drug resistance. Several types of lncRNAs have been identified in resistant cancer cells, including *ODRUL*, *MALAT1*, and *ANRIL.* This review discusses the use of natural products as natural inhibitors of P-gp expression. *In silico* analysis showed that Delphinidin and Asparagoside-f are the most significant natural product inhibitors of p-glycoprotein-1 to overcome resistance. Our findings could open new hope in minimizing the immorality of chemoresistance and improving the outcome of several types of cancers.

## Glossary

EMT: Epithelial-to-mesenchymal transition
lncRNA: Long non-coding RNA
P-gp: P-glycoproteins
ABC: ATP-binding cassette
MRP1: Multidrug resistance protein 1
MDR1: Multidrug resistance gene 1
CSCs: Cancer stem cells
ECM: Extracellular matrix
ROS: Reactive Oxygen Species
TGF-β: Transforming growth factor-beta
PM: Plasma membrane
PS: Phosphatidylserine
SLC: Several transporters named carriers
EGFR: Epidermal growth factor receptor
OATP1B3: Solute carrier organic anion transporter family member 1B3
OCT6: Organic cation transporter-6
ERP29: Endoplasmic reticulum protein 29
ID4: Inhibitor Of DNA Binding 4
MGMT: Methylated-DNA-protein-cysteine methyltransferase
ETS: ETS proto-oncogene 1
PD-L1: Programmed Cell Death Ligand 1
DNMT1: DNA methyltransferase 1
HCC: Hepatocellular carcinoma
TGBI: Transforming Growth Factor Beta Induced
ER-α: Estrogen Receptors Alpha
PEBP4: Phosphatidylethanolamine binding protein 4
RKIP: RAF-kinase inhibitor protein
DDR: DNA damage repair
MGMT: O-6-Methylguanine-DNA Methyltransferase
DNA-PK: DNA-dependent protein kinase
DSBs: Double-strand break repair machinery
eATP: Extracellular ATP
ABCG2: Adenosine triphosphate-binding cassette superfamily G member 2
MEK1/2: Mitogen-activated protein kinase 1/2
Raf- 1: RAF proto-oncogene
ERK: Extracellular signal-regulated kinase '
PDAC: ancreatic ductal adenocarcinoma
ODRUL: OS doxorubicin resistance-related upregulated lncRNA
HOTTIP: HOXA transcript at the distal tip
HOT AIR: HOX antisense intergenic RNA
LINC-ROR: Long Intergenic Non-Protein Coding RNA, Regulator of Reprogramming
CCAL: Colorectal cancer-associated lncRNA
MALAT1: metastasis-associated lung adenocarcinoma transcript 1
ANRIL: Antisense Noncoding RNA in the INK4 Locus
MRUL: MDR-related and upregulated lncRNA
EVs: Extracellular vesicles
Linc-VLDLR: Long intergenic non-coding RNA VLDLR
SGK1: Serum- and Glucocorticoid-Regulated Kinase 1
miRNA: microRNAs
XIST: X-inactive specific transcript
LINC00518: Long intergenic nonprotein coding RNA 518
BLACAT1: Bladder cancer-associated transcript-1
STAT1: Signal transducers and activators of transcription
